# Fracture Resistance of Premolars Restored by Various Types and Placement Techniques of Resin Composites

**DOI:** 10.1155/2012/973641

**Published:** 2012-05-14

**Authors:** Horieh Moosavi, Mahsa Zeynali, Zahra Hosseini Pour

**Affiliations:** ^1^Dental Material Research Center, Faculty of Dentistry, Mashhad University of Medical Sciences, Mashhad 91735, Iran; ^2^Department of Operative Dentistry, Mashhad Dental School, Mashhad University of Medical Sciences, Mashhad 91735, Iran

## Abstract

To verify the fracture resistance of premolars with mesioocclusodistal preparations restored by different resin composites and placement techniques. Sixty premolars were randomly divided into two groups based on type of composite resin: Filtek P60 or Nulite F, and then each group was separated into three subgroups: bulk, centripetal, and fiber insert according to the type of placement method (*n* = 10). Single-bond adhesive system was used as composite bonding according to the manufacturer's instructions. Specimens were restored in Groups 1, 2, and 3 with Filtek P60 and in Groups 4, 5, and 6 with Nulite F. After being stored 24 hours at 37°C, a 4 mm diameter steel sphere in a universal testing machine was applied on tooth buccal and lingual cusps at a cross-head speed of 5 mm/min until fracture occurred. Groups 3 and 6 showed higher fracture resistance than Groups 1, 2, 4, and 5. Among the placement techniques, the fiber insert method had a significant effect, but the type of composite was ineffective. The insertion technique in contrast to the type of material had a significant influence on the fracture resistance of premolar teeth.

## 1. Introduction

Dental restorative composites have been widely used over the past decade to restore posterior teeth. Occlusal wear and secondary caries are the predominant causes of failure in direct posterior composite fillings. However, fracture has also been reported to be a common reason for replacement [1]. Mesioocclusodistal cavity preparation brings about a significant reduction in tooth strength due to the loss of marginal ridges and microfractures caused by applied occlusal forces [2, 3]. Occlusally applied loads may tend to force cusps apart and in teeth with wide Class II cavities, a fracture of the cusps occurs as a result of fatigue of the brittle tooth structure by propagation of microcracks under repeated loading [4]. The introduction of composites and dentinal adhesives has been a significant contribution to the fracture resistance of teeth because it can reinforce the dental structure as a result of bonding to the tooth; in addition, the adhesive type has a significant effect on the fracture resistance [5]. The clinical performance of the newer dental composites has been significantly improved over the past decade to provide adequate strength and resistance in order to withstand the forces of mastication and provide less polymerization shrinkage and better cure depth. Nevertheless, the relatively high brittleness and low fracture toughness of current dental composites still remain a problem in stress-bearing posterior restorations [1]. A restored tooth tends to transfer stresses differently than an intact tooth [2], and the filling technique and cavity size have important effects on the bond strength of composite in the preparation [6, 7]. Moreover, adhesive restorations better transmit and distribute functional stresses across the bonding interface and have the potential to reinforce weakened tooth structure [8–10]. Polymerization of composites can cause deformation on the surrounded tooth structure resulting in microcracks which predispose the tooth to fracture [11]. In contrast to incrementally technique, if the preparation is bulk-filled with a single composite increment, the resulting high C-factor can further increase shrinkage stress [12]. Fracture resistance is one of the most important characteristics of dental materials. It depends on material resistance to crack propagation from its internal defects. These cracks can result in microscopic fractures of the restoration margins or bulk fracture of the filling [13]. Indirect ceromer inlays offered greater resistance to fractures compared with the intact tooth, but the fracture resistance of teeth resorted with Class II resin composites was not significantly different from that of ceromer [14]. Adhesive inlay restorations, irrespective of the type of composite resin and light-activation technique, restored the fracture resistance of intact teeth [15]. Reinforcing with short fibers has been revealed to control the polymerization shrinkage stress and microleakage compared with conventional composite resins [1]. Placement of resin composite which is reinforced with buccolingually oriented polyethylene fibers in endodontically treated teeth is a more promising technique than the older ones to restore the wide cavities [16]. The null hypothesis tested was that the type of composite and placement technique would have no effect on the fracture resistance of restored premolar teeth.

## 2. Materials and Methods

In this *in vitro* study, 60 recently extracted intact maxillary premolars, without caries, restoration, cracks, and fracture were collected and placed in 10% formalin solution for disinfection. To simulate periodontium, root surfaces were dipped into melted wax to a depth of 2 mm below the C.E.J to produce a 0.2-to-0.3 layer, and then mounted in polyvinyl plastic cylinders with self-cure acryl 2 mm below the C.E.J. Each tooth was removed from the acryl, and the wax spacer was removed from the root and acryl surfaces. Polyether was placed into the residual space, and teeth were reinserted into the cylinders. Thus, the periodontal ligament was simulated to some extent. For all specimens an operator prepared Class II cavities with a 2 ± 0.2 mm pulpal depth, 1.5 ± 0.2 mm gingival width, 2 ± 0.2 mm axial height, parallel proximal walls with 3 ± 0.2 mm buccolingual width and occlusal isthmus width one-third of the intercuspal distance. For better harmony among the cavities, a single periodontal probe was used as a guide, and no bevel was performed except for the axiopulpal line-angles. A bur was used to cut four teeth. Materials, compositions, and manufacturers are listed in Table 1.

Specimens were first divided into two groups of thirty according to the type of composite: Filtek P60 (3 M ESPE Dental Products) and Nulite F (BDT, NSW, Australia). Each group was then divided into three subgroups of ten according to the placement technique.

**Figure 1:**
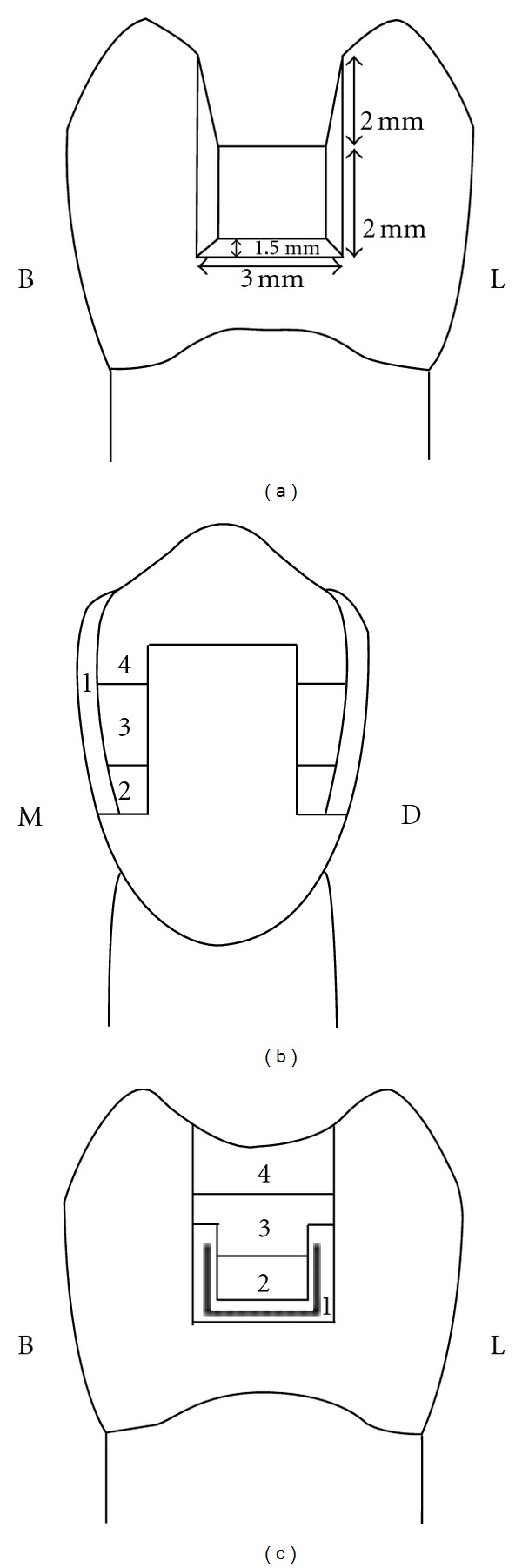
Various placement techniques in experimental groups from buccolingual (BL) or mesiodistaln (MD) view: bulk (a), centripetal (b), and fiber insert (c).

**Figure 2:**
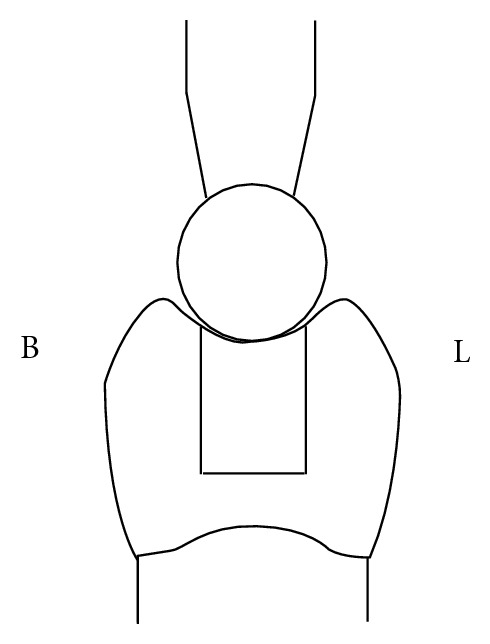
Schematic representation load cell on specimens in buccolingual (BL) view.

Bulk technique: cavities were filled with a single increment to restore the final contour and occlusally light-cured for 80 seconds (Figure 1(a)). Centripetal technique: the first increment of composite resin was applied on the gingival floor of the proximal box and packed near the axial wall causing the composite to climb upward in contact with the inner surface of the matrix band. This increment was light-cured, and subsequent layers (2 mm thick) were placed horizontally from the gingival floor toward the occlusal surface to fill the preparation. Each increment was light-cured for 40 seconds (Figure 1(b)). Horizontal incremental with fiber insert: first a composite layer of less than 1 mm thick was placed on the gingival floor. Before curing, a 3 mm piece of fiber insert was condensed in the composite resin to completely contact the gingival floor and the matrix band. Almost 2 mm of each proximal box was restored with composite impregnated fiber. This layer was cured occlusally for 40 seconds. The remainder of the cavity was filled with horizontal increments, and each was cured for 40 seconds (Figure 1(c)). The proportion of the fiber to composite was approximately one-third of each proximal box. In all groups, postcuring was done from the buccal and lingual for 40 seconds after removing the matrix band therefore, Groups 1, 2, and 3 were restored with Filtek P60 and with bulk, centripetal, and horizontal incremental with fiber insert techniques, respectively, and Groups 2, 4, and 6 were restored with Nulite F with bulk, centripetal, and horizontal incremental with fiber insert techniques, respectively. To simulate the clinical conditions, metal matrix bands and the “Tofflemier” matrix holder were used. Single bond (3 M ESPE, St. Paul, MN, USA) was applied in all specimens following the manufacturer's recommendations, and light curing was done with Optilux 500 (Demetron-Kerr, Orange, CA, USA) with a light intensity of 500 mW/cm^2^. Ten minutes after the restorative procedure, restorations were finished with a 12-blade finishing bur and polished with rubber points in a low-speed handpiece. The specimens were stored in 37°C distilled water, and then the fracture resistance test was conducted in an instron testing machine (Zwick, Germany). A 4 mm diameter steel sphere was applied on the buccal and lingual cusps of the tested teeth at a cross-head speed of 5 mm/min until the fracture occurred (Figure 2). The force, at which the tooth fractured, was recorded in Newton as the fracture resistance.

## 3. Results

**Figure 3:**
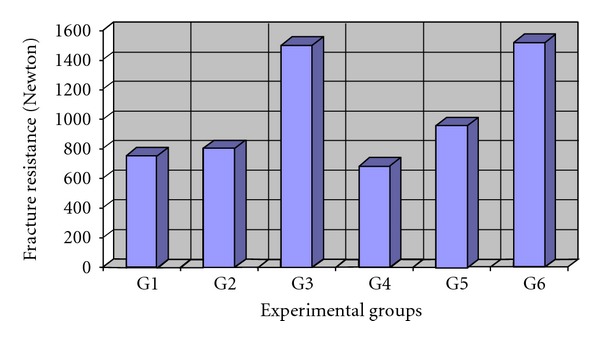
Mean value of fracture resistance in experimental groups.

Fracture strength results for experimental groups are displayed in Table 2 and Figure 3. According to this Table Group 6 and Group 4 had the maximum (1517.34) and minimum (682.90) of the fracture resistance values, respectively. First, one-way ANOVA indicated a significant difference in fracture resistance values of the test groups. The Duncan test revealed that significant difference exists between mean values of Groups 3 and 6 with the others. No significant difference was observed among Groups 1, 2, 4, and 5. In analysis of the effect of placement technique and type of composite, the two-way ANOVA indicated that only the placement technique significantly affected the fracture resistance (*P* = 0.018), not the composite type (*P* = 0.662). The interaction effect of composite type and placement technique did not have a significant effect on the fracture resistance (*P* = 0.58). The Duncan test demonstrated that the fiber insert technique lead to the highest fracture resistance which was significantly different from bulk and centripetal techniques.

## 4. Discussion

In this study, the fracture resistance of the groups restored with fiber insert technique was significantly higher than the other two techniques. These findings were similar to the previous study in which they compared the fracture resistance of root-filled molars restored with bulk, a low-viscosity composite liner, and fiber insert techniques [8]. Stress transfer from the polymer matrix to fibers is essential for a fiber to be effective in reinforcing polymers [1, 17]. This is achieved if the fibers have an equal or greater length than the critical fiber length [1]. Fiber critical length depends on factors such as the shear strength of the matrix, strength of the interfacial bond, and the tensile strength of the fiber [17]. In the study, in order to obtain a polishable and tooth-coloured surface [18], fiber length was equal to the buccolingual dimension of the proximal box (3 mm) and was parallel to the axial wall to restore the 2 mm of the lost proximal height, which was greater than the fiber critical length. The fiber function is based on supporting the surface composite layer and working as a crack stopper [1]. Polyethylene fiber is believed to create a change in the stress dynamics at restoration/adhesive interface. Also, fibers replace part of the composite, resulting in a decrease in the overall volumetric contraction of the composite and blunt the crack and can act as a barrier to crack propagation and decreasing the shrinkage stress [19, 20]. It has been reported that shear bond strength of resin composite to fiber-reinforced substrates depends on the load to fiber direction, and it is higher when the load direction corresponds to the fibers direction [21]. So, a reason for the higher fracture resistance in the fiber insert groups seems to be the buccal-lingual fiber orientation with the same direction of the applied load which has a splinting effect on the proximal walls in order to prevent separation of cusps under occlusal loading. According to the anisotropic character of the fibers, this kind of orientation permits maximum loading [20]. Already no significant effect was reported for the fiber in composite resin restorations [22]. Using fiber insert for Class II composite resin restorations caused significantly reduction in microleakage [23]. In a previous study, the fracture resistance of premolars restored with three forms of composite resins, beta quartz inserts, horizontally and obliquely layered was compared and observed the maximum fracture resistance in the oblique-layered method. They demonstrated that beta quartz inserts act as mega filler, thereby reducing the polymerization shrinkage and resulting in a higher fracture resistance compared with the horizontally layered technique [2]. These findings are somewhat consistent with the present study that observed a higher fracture resistance in fiber insert groups than in centripetal and bulk methods. Present results confirmed that various placement technique of composite resin had essential role for improving and modifying of shrinkage stresses [24]. No significant difference was observed between the fracture resistance of centripetal and bulk placement method. Considering the centripetal technique as a layering method, we expected a higher fracture resistance than the bulk technique. This was in contrast with one study which reported that resin composites fabricated by incremental layering create low-fracture toughness planes the same as bulk-cured ones; whereas for the microfilled composite resin, this effect was not observed. Therefore, the study concluded that the direction of layering should be adjusted in relation to the occlusion, and the way the force would be applied to the restoration [25]. Although the centripetal technique did not have a significant difference with the bulk technique in fracture resistance, there are still some advantages for this method, such as facilitation of a Class II buildup, establishment of a proper proximal contact, and provision of adequate light exposure for polymerization [26]. In this study, no significant difference was observed between the fracture resistances of specimens restored with Filtek P60 or Nulite F. According to the higher percent of volumetric filler content in Nulite F (71%) than Filtek P60 (61%), a superior fracture resistance was expected for Nulite F. The fracture toughness of BIS-GMA resin short-glass fiber composites with filler contents of 40, 50, 60, and 70% was measured in a study, and the results showed that the compressive strength was dependent on the percent of filler content, and the highest fracture resistance was obtained at the 50% filler content [27]. Nulite F is fiber-reinforced composite containing short-fiber fillers. The properties of the fibers depend on the load direction subjected to them and fiber distribution type in this composite is not uniform and can be partly explained because of the fiber lengths is well below of the critical fiber length; therefore, lack of significant difference between fracture resistances of the two composites could be justified. No comparative study on Filtek P60 and Nulite F was found but, in a clinical study [28], fracture resistance and durability of fiber-reinforced composites was similar to other resin composites and SEM assessment of the fracture mode of resin composites showed that crack formation occurred at the interface between the fiber fillers and the resin matrix representing the poor bond between fiber and matrix. In the afore-mentioned study Nulite F represented a lower 6-year clinical performance than another fiber-reinforced commercial composite resin [28]. This study was conducted on premolar teeth, and fracture resistance was tested shortly after the restoration. However, there are some differences between induced fracture variables in oral cavity and *in vitro* studies which are included; the presence of thermal and chemical factors, physical, aging, fatigue stresses, variations of magnitude, speed, and directions of forces that related to the type of each individual occlusion. Stress applied to the teeth and restorations is generally cyclic rather than being isolated and impact, so, with regard to the design of the load test, next step can be to apply dynamic loading. Further investigation is necessary to evaluate the *in vivo* behavior of these materials and techniques on posterior restorations.

## 5. Conclusions

Within the limits of this study, it can be concluded that.

Inserting a polyethylene fiber in composite restorations significantly increased the fracture resistance.

Type of composite (P60 or Nulite F) did not make a significant difference in the fracture resistance of premolars with composite restorations.

## Figures and Tables

**Table 1:** Chemical composition and manufacture of bonding agent and restorative materials used in this study.

Material		Composition	Manufacture
Bonding agent	Single bond	Bis-GMA, HEMA, dimethacrylates, polyalkenoic copolymer, ethanol, water, and photoinitiator	3 M ESPE St. Paul, MN, USA
	Filtek P60	Silane-treated ceramic 61% V, BISEMA6, UDMA, BISGMA, and TEGDM	3 M ESPE dental products St. Paul, MN, USA
Restorative materials	Nulite F	Bis-GMA and microrod filler 71% V (fiber-reinforced composite)	BDT-biodental technologies Pty limited, Australia
	Fiber insert	Ribbond-THM (polyethylene fiber)	Ribbond-THM, Seattle, WA, USA

Bis-GMA: bisphenol-A-glycidyl methacrylate, HEMA: hydroxyethyl methacrylate, BISEMA6: bisphenol A polyethylene glycol diether dimethacrylate, UDMA: diurethane dimethacrylate, and TEGDMA: triethylene glycol dimethacrylate.

**Table 2:** Means ± standard deviation, minimum, and maximum in Newton for experimental groups.

Group	*n*	Description of group	Means ± SD	Minimum	Maximum	dt
1	10	Filtek P60/bulk	754.14 (311.46)	210.11	1292.06	a
2	10	Filtek P60/centripetal	803.71 (248.20)	416.61	1196.99	a
3	10	Filtek P60/fiber insert	1498.61 (370.87)	1097.46	2122.10	b
4	10	Nulite F/bulk	682.90 (157.01)	447.74	935.14	a
5	10	Nulite F/centripetal	954.73 (281.21)	496.65	1312.15	a
6	10	Nulite F/fiber insert	1517.34 (530.89)	1055.06	2481.45	b

SD: standard deviation. Dt: Duncan's multiple range test for the different groups. Means with the same letter within each column are not significantly different at *P* = 0.05.
